# Environmental tobacco smoke exposure and non-syndromic orofacial cleft: Systematic review and meta-analysis

**DOI:** 10.18332/tid/163177

**Published:** 2023-06-12

**Authors:** Heba J. Sabbagh, Khlood K. Baghlaf, Hattan M. H. Jamalellail, Abdullah S. Bakhuraybah, Salem M. AlGhamdi, Omar A. Alharbi, Khalid M. AlHarbi, Mona H. A. Hassan

**Affiliations:** 1 Department of Pediatric Dentistry, Faculty of Dentistry, King Abdulaziz University, Jeddah, Saudi Arabia; 2 Faculty of Dentistry, King Abdulaziz University, Jeddah, Saudi Arabia; 3 Primary Health Care, Jizan Department, Ministry of National Guard Health Affairs, Jeddah, Saudi Arabia; 4 Department of Biostatistics, High Institute of Public Health, Alexandria University, Alexandria, Egypt

**Keywords:** cleft lip, cleft palate, orofacial cleft, passive smoking, environmental tobacco smoke

## Abstract

**INTRODUCTION:**

Environmental tobacco smoke (ETS) is associated with several congenital anomalies, including non-syndromic orofacial clefts (NSOFCs). This systematic review aimed to update the literature on the association between ETS and NSOFCs.

**METHODS:**

Four databases were searched up to March 2022, and studies that evaluated the association between ETS and NSOFCs were selected. Two authors selected the studies, extracted the data, and evaluated the risk of bias. Comparing the association of maternal exposure to ETS and active parental smoking with NSOFCs allowed for the creation of pooled effect estimates for the included studies.

**RESULTS:**

Twenty-six studies were deemed eligible for this review, of which 14 were reported in a previous systematic review. Twenty five were case-control studies, and one was a cohort study. In total, these studies included 2142 NSOFC cases compared to 118129 controls. All meta-analyses showed an association between ETS and the risk of having a child with NSOFC, based on the cleft phenotype, risk of bias, and year of publication, with a pooled increased odds ratio of 1.80 (95% CI: 1.51–2.15). These studies had a marked heterogeneity, which decreased upon subgrouping based on the recent year of publication and the risk of bias.

**CONCLUSIONS:**

ETS exposure was associated with more than a 1.5-fold increase in the risk of having a child with NSOFC, showing a higher odds ratio than paternal and maternal active smoking.

**TRIAL REGISTRATION:**

The study is registered on the International Prospective Register of Systematic Reviews database # CRD42021272909.

## INTRODUCTION

Smoking has been a controversial topic for decades; it remains one of the leading causes of lung cancer in men and breast cancer in women^1,2^. Smoking may be active or passive. According to the World Health Organization, active smoking is defined as smoking at least one cigarette a day. In contrast, passive smoking is the inhalation of tobacco smoke, also known as secondhand smoke or environmental tobacco smoke (ETS)^3^.

A recent study reported that ETS increased the risk of developing cardiovascular diseases by 28% and its associated mortality rate by 12%. Individuals affected by ETS are exposed to tobacco smoke at home, at work, and in public places^4^. ETS and active smoking have also been positively associated with congenital anomalies such as neural tube defects^5^, congenital heart defects^6^, and non-syndromic orofacial clefts (NSOFCs)^7-9^.

Syndromic orofacial clefts (OFC) are associated with structural or developmental defects, whereas NSOFCs are isolated and unrelated to other abnormalities^10^. This condition affects the quality of life; many patients with OFC develop depression, anxiety, lack of self-esteem^11^, speech defects, facial deformities, and several dental problems, including malocclusion^12^. The treatment of OFCs necessitates a multidisciplinary approach, with treatment ranging from infancy through late adolescence^13^.

Globally, the prevalence of NSOFCs is 1.25 per 1000 live births^8,14^. In 2004, a systematic review and meta-analysis, including 24 case-control studies, evaluated the association between maternal active smoking and the risk of having a child with NSOFC. They reported a modest dose-response effect for cleft lip with or without cleft palate^15^. In 2014, a systematic review and meta-analysis, including 14 case-control studies, evaluated the association between ETS and the risk of having a child with NSOFC and reported a positive odds ratio^8^. They also recommended further investigation to provide solid grounds for nicotine exposure^8^.

Since then, many studies have assessed the association between ETS and NSOFCs, and there is a need to update newly published evidence and evaluate the current evidence. Therefore, this systematic review and meta-analysis aimed to update the previous systematic review that pooled studies published up to 2013 by evaluating and comparing the evidence that investigates the association between maternal ETS exposure and NSOFCs in recent studies and published meta-analyses. In addition, it evaluates and compares paternal smoking with ETS exposure, which was not previously assessed.

## METHODS

The Preferred Reporting Items for Systematic Reviews and Meta-Analyses (PRISMA) guidelines^16^ were followed, and the findings were reported according to the PRISMA statement^17^.

### Information sources and search strategy

All relevant studies from 1980 to 2022 were identified. A comprehensive search of electronic databases, PubMed, Web of Science, Scopus, and ScienceDirect, between 2013 and March 2022. Studies published before 2013 were identified and recruited from the previous systematic review^8^. The search was not limited to studies published in English-language articles. A manual search of reference lists from identified published work and Google Scholar were also used to search for potentially eligible studies. Medical Subject Headings keywords were used to build a comprehensive search query. Repeated studies were detected and deleted using the EndNote reference manager (EndNote^®^ version 9, Niles Software, USA).

The following search terms were used: [(cleft lip) OR (cleft palate) OR (orofacial cleft)] AND [(passive smoking) OR (tobacco smoke pollution) OR (environmental tobacco smoke pollution) OR (smoking)].

Two researchers (AB and SG) were involved in the search strategy. All titles were independently reviewed by two researchers (OA and KH). All duplicates were excluded. Case-control, cohort, and cross-sectional studies that investigated the association between ETS and NSOFCs were included. Studies associated with syndromic OFCs, those that measured active smoking only, and those including genetic models were excluded.

### Eligibility criteria

Studies included in this review were selected in accordance with the PICO elements^18^: **P**articipants (studies assessing the etiology of NSOFCs), **I**ntervention or exposure (ETS), **C**omparison (healthy children without OFC), and **O**utcomes (NSOFCs).

### Study design

The inclusion criteria included case-control, cohort, and cross-sectional studies investigating the association between ETS and NSOFCs. Studies with a design other than the types mentioned in the inclusion criteria, those associated with syndromic OFCs, those that measured active smoking only, and those including genetic models, were excluded.

Other studies, such as editorials, letters to the editor, pilot studies, historical and literature reviews, *in vitro* studies, and descriptive studies, including case reports and case series, were also excluded.

### Study selection and data extraction

Two reviewers (KB and HJ) independently assessed the titles and abstracts of all the identified studies to determine if they met the inclusion criteria. The full-text articles of the selected studies were independently assessed by the same reviewers. Any disagreement between the two reviewers was resolved by consulting a third reviewer (HS). For studies performed on the same sample, studies with additional data were chosen. Two reviewers (KB and HS) assessed the selected articles using a standardized protocol, and the extracted data were recorded in a specific extraction datasheet. The extracted data included author names and citations, site, country, duration of data collection, study design, reported period of maternal exposure, total sample size, percentage of non-smoking mothers exposed to passive smoking and total non-smoking mothers, reported adjusted p-value, and adjusted odds ratio (95% confidence intervals [CI]) for passive smoking, maternal smoking and total sample size, and paternal smoking and total sample size.

*Quality assessment and the risk of bias*

Both cohort and case-control studies in this review were assessed independently using NOS^19^. The scale has a minimum score of 0 and a maximum of 9. It measures the selection of the cases, controls, and cohorts and how they represent the general population; the compatibility of cases, controls, and cohorts based on design and analysis; the exposure ascertainment of case controls; and the outcome of cohorts and the adequacy of their follow-up period. Studies that scored >6, 4–6, and ≤3 showed a low, moderate, and high risk of bias, respectively. Studies of moderate and high methodological quality (>5 stars) were included in the meta-analysis^8^. In case of any discrepancy between the two authors, the values were discussed until agreement. The level of agreement between the two authors was evaluated using the kappa score. Grading of Recommendations, Assessment, Development and Evaluations (GRADE) was used to summarize and assess the confidence of evidence and the strength of recommendations. It consists of five domains: risk of bias, inconsistency, indirectness, imprecision, and publication bias.

### Data synthesis

Data from the included studies were compiled. The data were organized according to author names and citations, site, duration of the data collection, study design, reported period of maternal ETS exposure, total sample size, percentage of mothers exposed to ETS with their respective p-values and odds ratios, percentage of maternal smoking and total sample size with their respective odds ratios, percentage of paternal smoking, and total sample size with their respective odds ratios, and risk of bias. If needed, a meta-analysis of the association between NSOFCs and ETS was performed.

Both quantitative and qualitative syntheses were performed wherever possible. Studies that compared the association of ETS with different cleft subtypes, including cleft lip (CL) and cleft palate (CP), were presented separately. Quantitative synthesis requires a minimum of two investigations. RevMan was used to conduct the meta-analysis (version 5.1; Nordic Cochrane Center, Cochrane Collaboration, 2001). Cochran’s test and Higgin’s I^2^ index were used to check for study heterogeneity. I^2^ statistic was classified into moderate heterogeneity (30% to 60%), substantial heterogeneity (50% to 90%) and considerable heterogeneity (75% to 100%)^20^. When there was a study’s heterogeneity, a random-effects model was conducted.

Sensitivity analyses based on subgroups were carried out according to the quality of the studies, the year of publication, and cleft types. Additionally, we assessed active parental smoking in the included studies and compared the results. The formal method of combining individual study data was the odds ratio for individual studies. Subgroup differences were tested using chi-squared. A funnel plot was used to visually assess the probability of small-study effects. Egger’s test was used to evaluate publication bias. The significance level was set at p<0.05.

Additionally, meta-regression analysis was performed using Meta-DiSc version 1.4 (http://www.hrc.es/investigacion/metadisc_en.htm) to assess the possible effects of the year of publication, the quality of the study, and the type of smoking on the association between ETS and NSOFCs. All variables entered in the model were binary.

## RESULTS

### Study selection

The search results from the databases yielded 1081 eligible titles. After the removal of duplicate results, only 821 articles remained. After screening the titles and reviewing the abstracts, only 21 full-text articles were obtained for comparison that met the inclusion and exclusion criteria. Of these, nine studies were excluded because of: a lack of specification regarding whether mothers were exposed to passive or active smoking (seven studies), focusing on the genetic effects of smoking on newborns (one study) and an overlapping population (one study) (Supplementary file Figure 1). Finally, 12 articles met the inclusion criteria for this systematic review and were suitable for inclusion in the qualitative synthesis (Supplementary file Table 1).

Additionally, 14 studies^7,9,15,21-30^ from the primary systematic review^8^ were included to update the review of this topic and study its effects over multiple decades. Totally, 26 studies were deemed eligible for this review; 25 were case-control studies^7,9,15,21-40^, and one was a prospective cohort study^41^. The data from the two centers were presented separately in a systematic review by Pi et al.^15,22,26,29,35,37,42^ (2018 A and B). To collect data on smoking, all of the included studies used self-reported questionnaires. Case-control studies were population-based, hospital-based^7,9,23,24,27,30,31,33,38,39^, or multi-hospital-based^8,11,32,33^ (Table 1).

**Table 1:** Characteristics of studies

*Authors Year Country*	*Study design*	*Cleft type*	ETS mothers* n/N (%)	*AOR (95% CI)*	*Maternal smoking n/N (%)*	*AOR (95% CI)*	*Paternal smoking n/N (%)*	*AOR (95% CI)*	*Confounding variables*
Beaty et al.^21^ 2001 US	Case-control	NSOFC	24/107 (22.4)		27/171 (15.8)		-	-	Smoking, alcohol use, daily vitamin use, urinary tract infection
		CL/P	14/73 (19.20)	1.04 (0.067–1.62)	17/91 (19.0)	1.36 (0.68–2.72)			
		CP	10/34 (29.4)	1.17 (0.68–2.02)	10/44 (23.0)	1.74 (0.75–4.02)			
		Control	18/130 (13.8)		25/182 (13.7)				
Little et al.^15^ 2004 UK	Case-control Population-based	NSOFC	67/154 (43.5)		80/190 (42.1)				Maternal smoking
		CL/P	40/76 (52.6)	0.9 (0.5–1.7)	45/112 (40.0)	1.9 (1.1–3.1)	25/67 (37.0)	1.4 (0.7–2.9)	
		CP	27/78 (34.6)	1.1 (0.5–2.2)	35/78 (44.9)	2.3 (1.3–4.1)	11/24 (45.0)	2.2 (0.8–5.9)	
		Control	111/189 (58.7)		59/189 (31)		28/119 (23.5)		
Honein et al.^22^ 2007 US	Case-control Population-based	NSOFC	235/1227 (19.1)				-	-	Folic acid exposure, alcohol use, maternal smoking
		CL/P	147/699 (21.0)	1.0 (0.8–1.3)	200/1461 (13.6)	1.4 (1.1–1.7)	-	-	
		CP	88/528 (22.0)	1.1 (0.8–1.4)	92/1461 (6.2)	1.2 (0.9–1.6)			
		Control	554/2699 (20.5)		679/3390 (20.0)				
Chevrier et al.^40^ 2008 France	Case-control	NSOFC	97/173 (56.1)	1.8 (1.2–3.4)	27/171 (15.8)	1.1 (0.7–1.9)	-	-	Maternal dietary folate intake, alcohol consumption, maternal smoking
		CL/P	65/119 (54.6)			1.0 (0.5–2.0)			
		CP	32/54 (59.3)			1.0 (0.3–3.3)			
		Control	70/167 (41.9)	-	25/182 (13.7)	-	-	-	
Lie et al.^27^ 2008 Norway	Case-control Hospital-based	NSOFC	90/334 (26.9)	1.05 (0.55–2.00)	239/432 (55.0)	0.81 (0.45–1.44)	-	-	Cigarette smoking, folic acid supplement, dietary folate, multivitamins, alcohol use
		CL/P	58/210 (27.6)			1.82 (0.98–3.39)			
		CP	32/124 (25.8)			0.29 (0.04–2.26)			
		Control	106/520 (20.4)	-	243/763 (31.8)	-	-	-	
Leite and Koifman^24^ 2009 Brazil	Case-control Hospitalbased	NSOFC	166/274 (60.6)	1.48 (1.09–2.01)	51/274 (18.60)	1.28 (0.87–1.97)	59/274 (21.6)	1.02 (0.75–1.52)	Maternal smoking, alcohol use
		CL/P		1.39 (1.01–1.98)		1.59 (1.04–2.44)		1.17 (0.80–1.75)	
		CP		1.67 (0.90–3.11)		0.82 (0.34–1.79)		0.58 (0.19–1.27)	
		Control	281/548 (51)	-	88/548 (16.10)	1.43 (1.25–1.64)	118/548 (21.4)	-	
Wang et al.^29^ 2009 China	Case-control Population-based	NSOFC	168/586 (28.7)	2.05 (1.47–2.87)	12/344 (2.0)	1.5 (0.52–4.36)	178/334 (30.4)	1.11 (0.82–1.51)	Maternal illness, medication use, maternal smoking, toxic exposures, pesticides, alcohol, radiation therapy
		Control	192/1172 (16.4)	2.05 (1.47–2.87)	16/1172 (1.3)	1.5 (0.52-4.36)	330/1172 (28.2)	1.11 (0.82–1.51)	
Jianyan et al.^23^ 2010 China	Case-control Hospital-based	CL/P	121/200 (60.5)	1.72 (1.08–2.74)	-	-	105/200 (52.5)	1.04 (0.65–1.67)	Maternal smoking, multivitamins
		Control	87/200 (43.5)	1.72 (1.08–2.74)	-	-	91/200 (45.5)	1.04 (0.65–1.67)	
Li et al.^26^ 2010 China	Case-control Population-based	CL/P	59/88 (67.0)	2.0 (1.2–3.4)	-	-	-		Maternal flu or fever in early pregnancy, folic acid
		Control	348/651 (54.0)	2.0 (1.2–3.4)	-	-	-	-	
Zhang et al.^30^ 2010 China	Case-control Hospital-based	NSOFC	224/323 (69.3)		14/300 (4.6)				
		CL	79/106 (74.5)	3.71 (1.46–9.40)	4/86 (4.7)	7.00 (1.44–34.13)	40/86 (46.5)	14.64 (4.11–52.13)	
		CP	49/77 (36.3)	2.97 (1.32–7.79)	0/77 (0)	<0.01 (<0.01–999.9)	41/77 (53.2)	37.88 (10.5–36.43)	
		CLP	96/140 (68.6)	1.09 (0.41–2.93)	10/140 (7.2)	5.12 (1.30–20.12)	79/140 (56.4)	33.19 (10.5–04.87)	
		Control	169/454 (37.2)	–	6/454 (1.3)		17/454 (3.7)	-	
Jia et al.^7^ 2011 China	Case-control Hospital-based	NSOFC	402/713 (56.2)		18/713 (2.5)		435/713 (61.0)		Multivitamins supplement, maternal folic acid use, maternal calcium supplement, folic acid, alcohol
		CL⁄P	302/537 (56.2)	9.23 (5.96–14.28)	15/537 (2.7)	3.15 (0.71–13.88)	325/537 (60.5)	1.92 (1.40–2.62)	
		CP	100/176 (56.8)	9.45 (5.73–15.60)	3/176 (1.70)	1.90 (0.31–11.49)	110/176 (62.5)	2.09 (1.40–3.13)	
		Control	27/221 (12.2)	-	2/221 (0.9)	-	98/221 (44.3)	-	
Li et al.^25^ 2011 China	Case-Control	NSOFC	69/162 (42.6)	3.44 (2.24–5.27)	-	-	-	-	Maternal vitamin intake, alcohol use
		Control	54/204 (17.4)	-	-	-	-	-	
Mirilas et al.^28^ 2011 Greece	Case-Control Hospital-based	CL/P	34/35 (45.7)	1.81 (0.69–4.74)	6/35 (17.1)	0.82 (0.24–2.76)	22/35 (62.8)	1.26 (0.48–3.30)	Disease and drugs, exposure to environmental pollutants, exposure to chemical contaminants
		Control	25/3531 (31.4)	-	7/35 (20.0)	-	20/35 (57.1)	-	
Taghavi et al.^9^ 2012 Iran	Case-control Hospital-based	CL/P	113/300 (37.7)	0.613 (0.43-0.87)	7/300 (2.3)	0.516 (0.34–3.93)	-	-	Supplemental vitamin, folic acid use, radiation exposure, maternal smoking
		Control	80/300 (26.7)	-	5/300 (2.0)	-	-	-	
Hao et al.^34^ 2015 China	Case-control Multi-hospital	NSOFC	285/499 (57.11)						Medication use, maternal smoking, maternal alcohol
		CL/P	214/362 (59.1)	2.52 (1.90-3.33)	26/362 (7.2)	1.25 (0.72–2.17)	218/362 (60.2)	2.17 (1.65–2.87)	
		CP	71/137 (51.8)	1.87 (1.28–2.75)	9/137 (6.6)	1.14 (0.52–2.47)	82/137 (59.9)	2.14 (1.45–.15)	
		Control	175/480 (36.5)		28/480 (5.8)		197/480 (41.0)		
Sabbagh et al.^8^ 2015 Saudi Arabia	Case-control 11 Multi-hospital	NSOFC	45/204 (22.0)	1.18 (0.75–1.87)	6/204 (2.9)	0.6 (0.2–1.7)	74/204 (36.3)	1.01 (0.69–1.48)	Maternal medication use and illness, maternal supplements use, maternal stress, maternal domestic environmental exposure
		CL	10/77 (13.0)	0.64 (0.31–1.34)	2/77 (2.7)	0.71 (0.15–3.37)	17/77 (22.1)	0.51 (0.28–0.92)	
		CLP	21/74 (28.4)	1.68 (0.93–3.06)	4/74 (4.1)	0.99 (0.27–3.71)	33/74 (44.6)	1.38 (0.81–2.33)	
		CP	14/53 (26.4)	1.52 (0.76–3.03)	0/53 (0)		24/53 (45.3)	1.14 (0.78–2.58)	
		Control	47/244 (19.3)		10/244 (4.1)		90/244 (36.9)		
Hoyt et al.^35^ 2016 US	Case-control Population-based	NSOFC	148/1102 (13.4)	1.25 (1.09–1.04)					Maternal alcohol, prepregnancy body mass index, folic acid exposure, multivitamins
		CL	39/290 (13.4)	1.41 (1.12–1.81)					
		CLP	62/450 (13.8)	1.16 (0.95–1.51)					
		CP	47/362 (12.9)	1.31 (1.26–1.63)	-	-	-	-	
		Control n=3324	369/3324 (11.1)						
Kummet et al.^37^ 2016 Norway	Case-control Population-based	NSOFC	1914/9482 (21.1)	1.14 (1.02–1.27)	1030/14134 (7.2)	1.2 (1.11–1.46)			Active smoking exposure, alcohol use, supplements containing folic acid
		CL		1.14 (0.93–1.39)		1.52 (1.19–1.94)			
		CLP		1.11 (0.95–1.29)		1.18 (0.97–1.43)			
		CP		1.18 (1.00–139)		1.25 (1.01–1.55)			
		Control n=9626							
Mckinney et al.^38^ 2016 Thailand	Case-control Hospital-based	CL/P	41/95 (43.2)	6.52 (1.98– 21.44)	93/95 (98.0)	-	-	-	Maternal smoking, alcohol, folic acid, multivitamins
		Control	20/95 (21.1)	6.52 (1.98– 21.44)	92/95 (97.0)	-	-	-	
Dien et al.^32^ 2017 Vietnam	Case-control 3 Hospital-based	NSOFC	67/170 (39.4)	1.59 (0.50–5.09)	0/340 (0)	-	-	-	Maternal smoking, caffeine consumption, alcohol
		Control	43/170 (25.2)	-	-	-	-	-	
Goveas et al.^33^ 2017 India	Case-control multi- hospital based	CL/P	74/125 (59.2)	p=0.008, OR=1.97	-	p=0.498	-	-	Alcohol consumption, maternal smoking, multivitamins
		Control	53/125 (42.4)	-	-	-	-	-	
Junaid et al.^36^ 2018 India	Case-control 3 hospital based	NSOFC	24/50 (48.0)	2.46 (0.99–6.08)	1/50 (2.0)	p=1.00	20/50 (40.0)	p=0.41	Paternal alcohol use,paternal tobacco use, maternal tobacco exposure
		Control	12/50 (24.0)	2.46 (0.99–6.08)	1/50 (2.0)	-	16/50 (32.0)	-	
Pi et al.^42^ 2018 (2002–2011) China	Case-control Population- based	NSOFC	140/240 (58.3)						Maternal fever or flu
		CL/P	131/225 (58.2)	1.6 (1.2–2.2)	-	-	-	-	
		CP	9/15 (60.0)	p=0.003 1.6 (1.2–2.2)	-	-	-	-	
		Control	664/1420 (46.8)		-	-	-	-	
Pi et al.^42^ 2018 (2011-2016) China	Case-Control Population-based	CL/P	56/101 (55.4)	p=0.002 2.2 (1.4–3.6)					Maternal fever or flu
		Control n=561	173/561 (30.8)						
Altoe et al.^39^ 2019 Brazil	Case-control Hospital-based	NSOFC	32/150 (45.7)	1.98 (1.17–3.34)	13/150 (8.6)	2.04 (0.94–4.43)	-	-	Alcohol consumption, use of medication, diseases
		Control	38/300 (54.2)	-	15/300 (5.0)		-		
Chowchuen et al.^31^ 2021 Thailand	Case-control Hospital- based	CL/P	14/35 (40.0)	1.77 (0.52–6.04)	1/34 (2.86)	p=0.624	-	-	Alcohol intake, smoking, vitamin use, calcium, iron and folic acid
		Control	24/70 (34.29)	-	1/70 (1.4)	-	-	-	
Sato et al.^41^ 2021 Japan	Prospective- cohort	NSOFC	98/187 (52.4)		83/187 (44.4)				Psychological distress, maternal alcohol consumption, maternal active smoking, BMI, folic acid
		CL/P	82/146 (56.1)	1.49 (0.93–2.39)	68/146 (46.5)	0.82 (0.34–1.99)			
		CP	16/41 (39.0)	-	15/41 (36.5)				
		Control	46566/94174 (49.4)	-	38228/94174 (40.5)				

* Non-smoking mothers exposed to environmental tobacco smoke (ETS). AOR: adjusted odds ratio. N: total sample.

### ETS and NSOFCs

The definition of maternal ETS exposure was similar in all the included studies. However, Hao et al.^34^ defined it as exposure to smoke of more than one cigarette per day, either at work or at home. No definition was found in the study by Junaid et al.^36^, whereas Dien et al.^32^ used a cutoff point of 15 minutes to count as exposure.

Most studies have used the first trimester as the measurement period for maternal passive smoking. However, the study by Honein et al.^22^ used a period of three months before pregnancy until birth: one year of pre-gestation along with the first trimester^24^, the first 28 weeks of pregnancy^29^, and the first month before pregnancy through the end of the first trimester^28,30^. Some studies used multiple periods of measurement, including one-year pre-gestation and the first trimester and three months pre-gestation and the first trimester^9,38^. Junaid et al.^36^ did not mention the measurement period in their study, whereas Pi et al.^42^ used the measurement period from the last menstrual period till the second trimester^41^. In this systematic review, we combined exposure, pre-gestation, and the first trimester in the meta-analysis.

### Study quality and risk of bias

The included studies were assessed by two authors, AB and SA, and the inter-rater agreement for the evaluation of the risk of bias was very good (Kappa score = 89). The Supplementary file Table 1 shows the included 11 studies distributed according to the NOS risk of bias scores. Out of these, only two were found to have a low risk of bias^37,40^. The remaining nine studies were found to have a moderate to high risk of bias^31-36,38,39,41,42^. This was mainly due to the absence of comparability and matching between cases and controls in many studies^31,33,35,36,42^. Furthermore, the studies showed bias in exposure ascertainment, as it was not possible for interviewers to be blinded to the cases or control status. The NOS descriptions and scores for the included 11 studies are presented in Supplementary file Table 1.

### Meta-analysis

**Figure 1:**
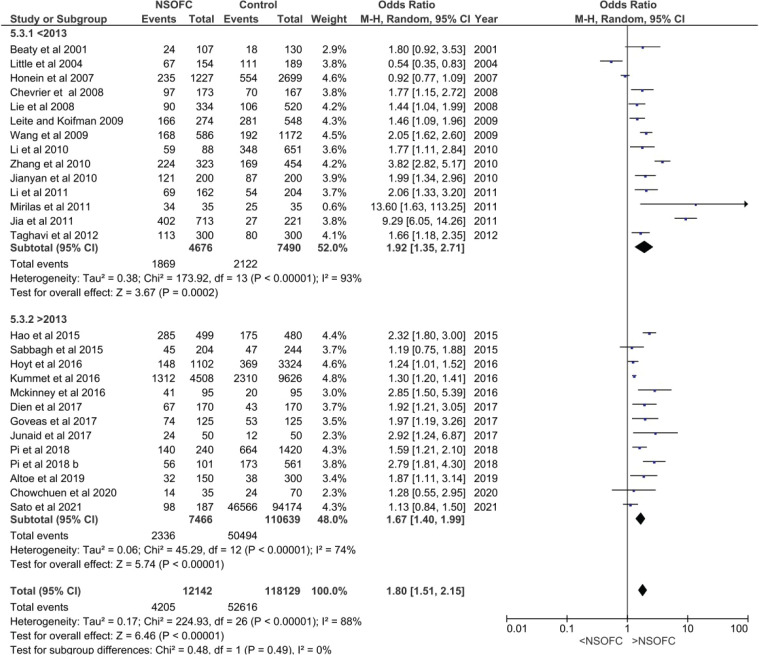
Forest plot for meta-analysis of the association between the risk of having an infant with NSOFC and maternal environmental tobacco exposure sub-grouped according to year of publication before and after 2013

Meta-analysis was conducted on 27 studies (considering the Pi et al.^42^ study to have two parts, A and B), which were then sub-grouped to assess sensitivity (Supplementary file Figures 2 and 4 to 8). The analysis included 12142 NSOFC cases and 118129 controls. There was a highly significant relationship between ETS and NSOFCs (p<0.01) with an increased odds ratio of having a child with NSOFC (OR=1.80; 95% CI: 1.51–2.15) (Table 2 and Figure 1).

**Table 2:** Results of meta-analysis subgrouping

*Subgroup analysis*		*OR (95% CI)*	*p*	*Heterogeneity I^2^ (%)*
**Publication year**	<2013	1.92 (1.35–2.71)	0.0002	93
	>2013	1.67 (1.40–1.99)	<0.001	74
**NSOFC types**	CL/P	1.85 (1.46–2.34)	<0.001	87
	CP	1.72 (1.13–2.63)	0.01	89
**Active smoking**	Maternal	1.51 (1.23–1.86)	<0.001	59
	Paternal	1.51 (1.11–2.06)	0.008	79
**Risk of bias**	Low	1.42 (1.17–1.71)	0.0003	81
	High	2.23 (1.65–3.01)	<0.001	88

*Year of publication*

The forest plot for the relationship between ETS and having a child with NSOFC, sub-grouped based on the year of publication, showed that studies published after 2013 had increased ETS odds ratio of having a child with NSOFC (OR=1.67; 95% CI: 1.40–1.99), similar to earlier studies. Even though the overall heterogeneity between studies was high (I^2^ =88%), it decreased to 74% in studies published after 2013 compared to those published before 2013. Nevertheless, there were no significant differences between the subgroups (p=0.49) (Figure 1).

*NSOFC phenotypes*

**Figure 2:**
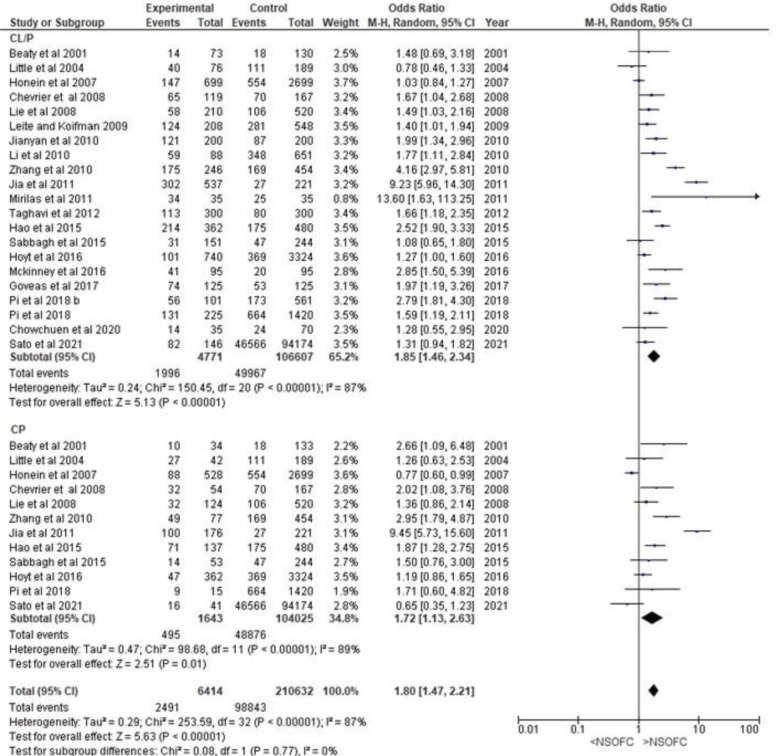
Forest plot for meta-analysis of the association between the risk of having an infant with cleft lip with or without cleft palate (CL/P) or cleft palate (CP) and its association with maternal environmental tobacco smoking

The forest plot for the relationship between ETS and the risk of having a child with CL or CP showed a highly significant correlation between ETS and both CL or CP and CP (p<0.001 and p=0.01, respectively), with increased odds ratio of 1.85 (95% CI: 1.46–2.34) for CL or CP and 1.80 (95% CI: 1.47–2.21) for CP. After removing the two studies, one with a high odds ratio^7^ and one with an extremely high CI^28^, the odds ratio remained high and significant (OR=1.57; 95% CI: 1.33–1.84 for CL or CP; and OR=1.44; CI: 1.31–1.75 for CP) (Figure 2).

Only three studies evaluated the CL or CP subphenotypes^30,35,40^. Only one of these studies had a low risk of bias^40^. Maternal ETS exposure showed a non-significant increase in the OR for both CL (OR=1.61; 95% CI: 0.54–4.82) and CP (OR=1.98; 95% CI: 0.98–4.01).

### Maternal and paternal active smoking

The forest plot for the meta-analysis of the association between the risk of having a child with NSOFC and parental active smoking among studies that evaluated ETS showed a significant association with an increased OR between maternal (OR=1.53; 95% CI: 1.23–1.88; p<0.001) and paternal active smoking (OR=1.51; 95% CI: 1.11–2.06; p<0.001) and NSOFCs. Among the studies that evaluated the paternal active smoking effect on NSOFCs, maternal ETS showed a higher OR (OR=2.21; 95% CI: 1.42–3.45). However, there was no significant difference between maternal ETS and paternal smoking subgrouping (p=0.17) (Supplementary file Figures 3 and 4).

### Sensitivity test

To demonstrate the stability and reliability of the meta-analysis results, a sensitivity analysis was conducted between different study subgroups according to cleft phenotype (Figure 2), the exclusion of studies with extreme results (Supplementary file Figure 6), the risk of bias (Supplementary file Figure 5) and the period of maternal ETS exposure (Supplementary file Figure 8). All meta-analyses showed consistent results of a significant association between maternal ETS exposure and an increased OR for the risk of having a child with NSOFC. However, none of the sub-grouped studies accounted for substantial heterogeneity between the studies. All meta-analyses showed significant heterogeneity of ≥75%.

### Evaluation of small-study effects

The funnel plots for all included studies that assessed the relationship between NSOFC phenotypes (CL or CP and CP) and passive smoking did not have the shape of a funnel. However, it was almost a symmetrical funnel plot around the central line, which indicates a publication bias (Supplementary file Figures 9 and 10). However, Egger’s test detected no publication bias.

### Stability of the evidence

The cumulative meta-analysis figure shows the stability of the evidence from 2011 to 2021. Regarding sufficiency (‘Are additional studies needed to establish the existence of the phenomenon?’), from the beginning of this cumulative meta-analysis, the 95% CIs around the OR included the final average effect size (ES) obtained at the end of the cumulative meta-analysis. Regarding stability (‘Will additional studies change the aggregate picture of the phenomenon?’), from the beginning of this cumulative meta-analysis, the mean ES appeared to be stable. Therefore, it would be difficult to argue that a subsequent study would alter the emergent picture of this effect beyond the evidence that the first few studies have produced (Supplementary file Figure 10).

### Meta-regression random effects model

A significant model implies that ES is associated with the variables (Table 3). There is a significant difference within groups, which shows that there may be more variables associated with ES. ES was lower for studies conducted after 2013 than for those before 2013. ES was greater in low-quality studies than in high-quality studies. Smoking, whether active or passive, did not affect ES after controlling for other variables in the meta-analysis model. R^2^ indicated that 33% of the heterogeneity was accounted for by the addition of predictors to the model compared to an ‘empty’ model. In other words, this represented the percentage of heterogeneity explained by group-level variables in the model (Supplementary file Figure 11).

**Table 3:** GRADE profile

*Quality assessment*							*Number of patients*		*Effect*	*Quality*
*Number of studies*	*Design*	*Risk of bias*	*Inconsistency*	*Indirectness*	*Imprecision*	*Other considerations*	NSOFC*	*Control*		
26	Case-control and cohort^a^	Serious^b^	Not serious^c^	Not serious^d^	Not serious^e^	Confounding^f^	11798	117985^g^	Pooled^h^	⊕⊕⊕◯ Moderate

Question: Is the environmental tobacco smoking associated with the risk of having an infant with NSOFC in all included twenty-six studies? Setting: general infant population.

* NSOFC (case-control studies); measured with standard indices; better indicated by lower values.

a Twenty-six case-control studies and one cohort. Sato et al. 2021 investigated the association between maternal ETS and the risk of having an infant with NSOFC in all included twenty-six studies. The OR ranged from 0.54 to 9.29.

b In Hoyt et al. 2016, Mckinney et al. 2016, Dien et al. 2017, Goveas et al. 2017, Junaid et al. 2018, and Pi et al. 2018 there was a serious risk of bias; therefore, there was a downgrading for risk of bias.

c There was no evidence of inconsistency. Most studies showed a significant association between NSIFC and ETS. Therefore, no downgrading was done for this inconsistency.

d Data were not downgraded for indirectness because all case-control studies were conducted worldwide.

e No downgrading for imprecision because all confidence intervals were narrow and no overlaps.

f No downgrading due to the plausible confounding was done; most studies controlled for the other confounding factors such as patient cooperation, isolation of the tooth and type of the teeth (upper or lower molars).

g Total number of infants from the 27 studies.

h Pooling of meta-analysis z=6.23, p=0.000001 with high heterogeneity.

## DISCUSSION

A systematic review conducted in 2015 assessed the association between maternal ETS exposure and NSOFCs that included studies published between 1980 and 2013^8^. However, paternal smoking in ETS studies and the association between ETS and CL/P subphenotypes (CL vs CLP) were not sufficiently discussed. Therefore, this systematic review was conducted to update the literature. This study consistently suggests a more than 1.5 increase in the risk of NSOFC phenotypes associated with paternal active smoking. A corresponding value of 2.21 was observed with ETS exposure.

In 2020, the World Health Organization highlighted a wide range of adverse health effects of nicotine exposure on infant and child development that result from ETS. They have urged protective policies directed toward smoke-free generations^43^. Maternal ETS exposure is associated with multiple birth defects and stillbirth^44^. ETS has been reported to cause fetal hypoxia, which leads to fetal growth retardation^45,46^. In this systematic review, the association and OR between maternal ETS exposure and having a child with NSOFC were significant. The difference between the outcomes of the studies published before and after 2013 was not significant. However, there was a small decrease in the OR (from 1.92 to 1.67), a smaller 95% CI range [from (1.35–2.71) to (1.40–1.99)], and heterogeneity (I^2^= 93% to 74%), which could be related to the recent improvement in study design and data collection method. Additionally, the studies published after 2013 showed less heterogeneity.

Furthermore, paternal smoking could be associated with having a child with NSOFC either directly by affecting sperm development or indirectly by increasing maternal ETS exposure^47-49^. Our findings suggest a possible association between ETS and NSOFCs (OR=2.21) that is stronger than that with active paternal smoking (OR=1.51) which could support the indirect effect of paternal smoking by increasing maternal ETS exposure. However, as the difference between the two associations was not significant, this suggestion needs further investigation to verify it.

In this systematic review, the OR of maternal ETS exposure and having a child with CL or CP was higher than that of having a child with CP (Figure 2). However, in the old systematic review, the OR of ETS and having a child with CP was similar to that of having a child with CL or CP^8^. Additionally, this study further assessed the NSOFC phenotype by including three articles investigating ETS association with CL, CLP, and CP formation. The study found an association between an increased risk of CL and CLP, though the available information was inadequate for reporting significant findings. Thus, further studies are needed to evaluate the effects of ETS on different NSOFC subphenotypes.

Our study supports the importance of implementing smoke-free legislation. In England and Northern Ireland, a study assessed the impact of smoke-free legislation on the prevalence of NSOFC. A reduction of 37% and 8%, respectively, in smoking was detected among active female smokers between 2000 and 2018^50^. Although they found no significant reduction in NSOFC prevalence, their results highlight the importance of public health measures, including smoke-free legislation in restaurants and prevention programs among pregnant females in controlling active smoking^51^.

The current worldwide response to the coronavirus disease 2019 (COVID-19) was a significant and widespread effect on stress and psychological conditions^52^. The COVID-19 pandemic has influenced the lifestyles of individuals, affecting their nicotine use and exposure^53,54^. However, a slight decrease in ETS was reported due to the lockdown^53^. This systematic review did not find any studies that evaluated nicotine exposure in NSOFCs after the COVID-19 pandemic. Therefore, future studies should evaluate this period.

### Limitations

Even though our meta-analysis included 26 articles, there were a few limitations. The heterogeneity between studies and the restricted high-quality case-control studies were the two main limitations. Thus, meta-analyses of some findings might lack adequate power and not allow accurate evaluation of heterogeneity with small-study effects and reporting biases. Combining evidence is also more challenging in the presence of different confounding variables, such as the frequency and distance of passive smoking. Moreover, there is still limited evidence supporting the effect of ETS on the development of different NSOFC sub-phenotypes and severity. These limitations and gaps in the literature highlight the need for well-conducted cohort studies that consider the definition of passive smoking and the evaluation of nicotine exposure using a validated, exact method instead of a subjective method like a questionnaire.

## CONCLUSIONS

There was a highly significant association between maternal ETS exposure and NSOFCs in children, indicating the importance of implementing smoke-free legislation and maternal pregnancy care. However, the included studies showed marked heterogeneity. Future case-control studies to examine the association between ETS exposure and NSOFCs should consider the definition of ETS and the evaluation of nicotine exposure using an objective measuring tool.

## Data Availability

The data supporting this research are available from the authors on reasonable request.
