# Supplementary material for: Environmental tobacco smoke exposure and non-syndromic orofacial cleft: Systematic review and meta-analysis
Source: Tob Induc Dis. 2023 Jun 12;21:76. doi: 10.18332/tid/163177 (PMC10258680; doi:10.18332/tid/163177)
Supplement: Supplementary file 1 [file TID-21-76-s1.pdf]

Supplementary Table 1. Summary of Newcastle-Ottawa Assessment scale of included studies.

Newcastle-Ottawa Assessment scale of eleven case-control studies				
Reference	Selection	Comparability	Exposure	Overall score
Hao et al 2015	***	**	*	6 stars
Sabbagh et al 2015	***	**	**	7 stars
Hoyt et al 2016	***	_	*	4 stars
Kummet et al 2016	***	**	**	7 stars
Mckinney et al 2016	**	**	*	5stars
Dien et al 2017	**	**	*	5 stars
Goveas et al 2017	**	_	*	3 stars
Junaid et al 2017	**	**	*	5 stars
Pi et al 2018	**	**	*	5 stars
Altoe et al 2019	-	*	**	3 stars
Chowchuen et al 2020	**	-	*	3 stars
Newcastle-Ottawa Assessment scale of Cohort studies				
Reference	Selection	Comparability	Outcome	Overall score
Sato et al 2021	***	*	*	5 stars

Supplementary Table 2. Egger's Regression-Based Test.

Parameter	Coefficient	Std. Error	t	p-value		95% CI
(Intercept)	.352	.2345	1.501	.146	-.131	.835
SE	1.103	1.0386	1.062	.298	-1.036	3.243

Random-effects meta-regression

SE Standard error of effect size

Supplementary Table 3. Meta-regression random effects (REML) model.

Covariate	Ref	Coefficient	SE	95% CI		t-value	P-value	VIF
Intercept		0.4888	0.1063	0.2741	0.7034	4.6	0	2.649
Year: >=2013	<2013	-0.6038	0.1754	-0.9581	-0.2495	-3.44	0.0013	1.756
Low quality	High quality	0.727	0.1742	0.3752	1.0788	4.17	0.0002	1.758
Passive smoking	Active smoking	-0.1257	0.1449	-0.4183	0.167	-0.87	0.3909	1.036

Statistics for Model 1

Test of the model: Simultaneous test that all coefficients (excluding intercept) are zero

F = 6.51, df = 3, 41, p = 0.0011

Goodness of fit: Test that unexplained variance is zero

Tau^2 = 0.1177, Tau = 0.3430, I^2 = 79.39%, Q = 198.98, df = 41, p = 0.0000

Comparison of Model 1 with the null model

Total between-study variance (intercept only)

Tau^2 = 0.1768, Tau = 0.4205, I^2 = 83.14%, Q = 261.03, df = 44, p = 0.0000

The proportion of total between-study variance explained by Model 1

R^2 analog = 0.33

Supplementary Figure 1. PRISMA 2020 flow diagram for updated systematic reviews which included searches of databases and registers only.

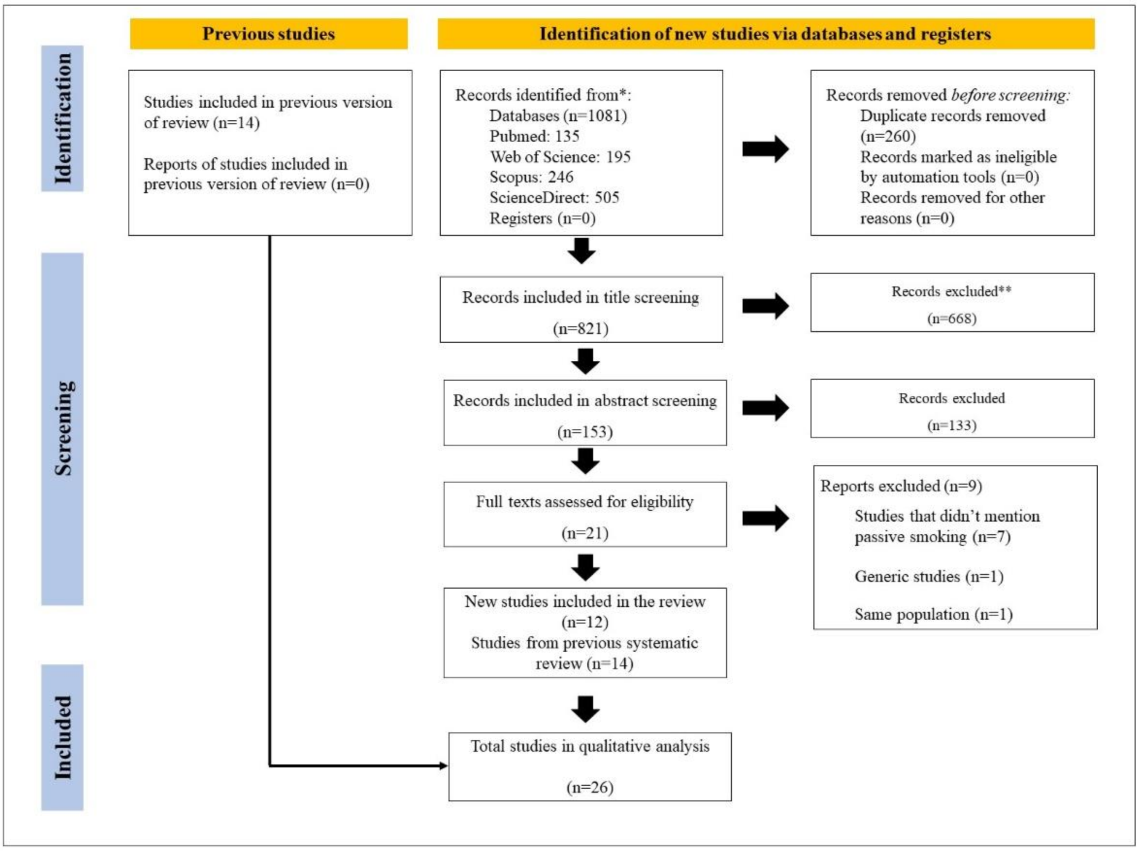

*Consider, if feasible to do so, reporting the number of records identified from each database or register searched (rather than the total number across all databases/registers).

**If automation tools were used, indicate how many records were excluded by a human and how many were excluded by automation tools.

From: Page MJ, McKenzie JE, Bossuyt PM, Boutron I, Hoffmann TC, Mulrow CD, et al. The PRISMA 2020 statement: an updated guideline for reporting systematic reviews. BMJ 2021;372:n71. doi: 10.1136/bmj.n71.

For more information, visit: http://www.prisma-statement.org/

Supplementary Figure 2. Forest plot for meta-analysis of the association between maternal environmental tobacco exposure and the risk of having an infant NSOFC sub-grouped according to CL/P phenotypes (CLP and CP).

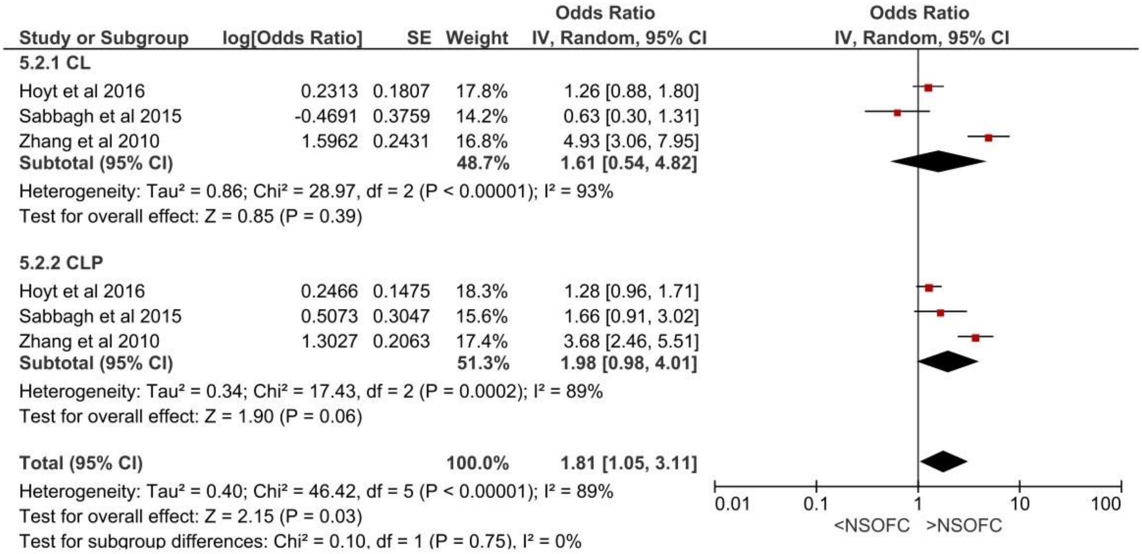

Supplementary Figure 3. Forest plot for meta-analysis of the association between the risk of having an infant with NSOFC and its association with maternal active smoking.

	NSOFC		Control			Odds Ratio	
Study or Subgroup	Events	Total	Events	Total	Weight	M-H, Random, 95% CI	Year
Beaty et al 2001	27	135	25	182	6.5%	1.57 [0.86, 2.85]	2001
Little et al 2004	80	190	59	189	8.9%	1.60 [1.05, 2.44]	2004
Honein et al 2007	352	1461	684	3390	13.1%	1.26 [1.08, 1.45]	2007
Chevrier et al 2008	27	171	25	182	6.6%	1.18 [0.65, 2.12]	2008
Lie et al 2008	239	432	243	763	11.7%	2.65 [2.08, 3.38]	2008
Leite and Koifman 2009	51	274	88	547	9.5%	1.19 [0.82, 1.74]	2009
Wang et al 2009	12	344	16	1172	4.9%	2.61 [1.22, 5.57]	2009
Zhang et al 2010	14	300	6	454	3.5%	3.66 [1.39, 9.62]	2010
Jia et al 2011	18	713	2	221	1.7%	2.84 [0.65, 12.32]	2011
Mirilas et al 2011	6	35	7	35	2.4%	0.83 [0.25, 2.77]	2011
Taghavi et al 2012	7	300	5	300	2.6%	1.41 [0.44, 4.49]	2012
Hao et al 2015	35	499	28	480	7.5%	1.22 [0.73, 2.03]	2015
Sabbagh et al 2015	6	204	10	244	3.1%	0.71 [0.25, 1.99]	2015
Mckinney et al 2016	93	95	92	95	1.2%	1.52 [0.25, 9.29]	2016
Junaid et al 2017	1	50	1	50	0.5%	1.00 [0.06, 16.44]	2017
Altoe et al 2019	13	150	15	300	4.8%	1.80 [0.83, 3.89]	2019
Chowchuen et al 2020	1	34	1	70	0.5%	2.09 [0.13, 34.48]	2020
Sato et al 2021	83	187	38228	94174	11.0%	1.17 [0.88, 1.56]	2021
**Total (95% CI)**		**5574**		**102848**	**100.0%**	**1.51 [1.23, 1.86]**	
Total events	1065		39535				

Heterogeneity: Tau² = 0.08; Chi² = 41.42, df = 17 (P = 0.0008); I² = 59%
Test for overall effect: Z = 3.92 (P < 0.0001)

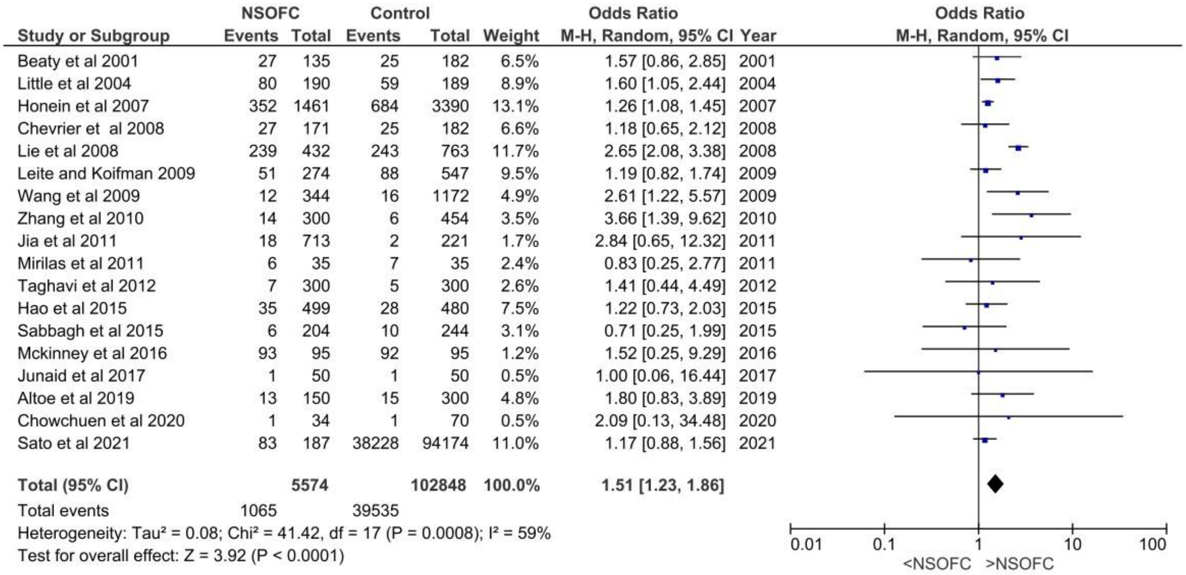

Supplementary Figure 4. Forest plot for meta-analysis of the association between the risk of having an infant with NSOFC and its association with paternal active smoking compared to maternal environmental tobacco smoking.

Study or Subgroup	NSOFC Events	NSOFC Total	Control Events	Control Total	Weight	Odds Ratio M-H, Random, 95% CI	Year
**4.1.1 Paternal smoking**							
Little et al 2004	36	91	28	119	5.0%	2.13 [1.17, 3.86]	2004
Wang et al 2009	178	334	330	1172	6.3%	2.91 [2.27, 3.74]	2009
Jianyan et al 2010	105	200	91	200	5.8%	1.32 [0.89, 1.96]	2010
Zhang et al 2010	110	167	325	537	5.9%	1.26 [0.88, 1.81]	2010
Jia et al 2011	435	713	98	221	6.1%	1.96 [1.45, 2.66]	2011
Li et al 2011	22	35	20	35	3.7%	1.27 [0.49, 3.31]	2011
Sabbagh et al 2015	74	204	90	244	5.9%	0.97 [0.66, 1.43]	2015
Hao et al 2015	218	362	82	137	5.8%	1.02 [0.68, 1.52]	2015
Junaid et al 2017	20	50	16	50	4.2%	1.42 [0.62, 3.22]	2017
**Subtotal (95% CI)**		**2156**		**2715**	**48.7%**	**1.51 [1.11, 2.06]**	
Total events	1198		1080				
Heterogeneity: Tau² = 0.16; Chi² = 38.26, df = 8 (P < 0.00001); I² = 79%							
Test for overall effect: Z = 2.65 (P = 0.008)							
**4.1.2 ETS**							
Little et al 2004	67	154	111	189	5.7%	0.54 [0.35, 0.83]	2004
Wang et al 2009	168	586	192	1172	6.3%	2.05 [1.62, 2.60]	2009
Jianyan et al 2010	121	200	87	200	5.8%	1.99 [1.34, 2.96]	2010
Zhang et al 2010	224	323	169	454	6.1%	3.82 [2.82, 5.17]	2010
Li et al 2011	69	162	54	204	5.7%	2.06 [1.33, 3.20]	2011
Jia et al 2011	402	713	27	221	5.7%	9.29 [6.05, 14.26]	2011
Sabbagh et al 2015	45	204	47	244	5.6%	1.19 [0.75, 1.88]	2015
Hao et al 2015	285	499	175	480	6.3%	2.32 [1.80, 3.00]	2015
Junaid et al 2017	24	50	12	50	4.0%	2.92 [1.24, 6.87]	2017
**Subtotal (95% CI)**		**2891**		**3214**	**51.3%**	**2.21 [1.42, 3.45]**	
Total events	1405		874				
Heterogeneity: Tau² = 0.41; Chi² = 105.00, df = 8 (P < 0.00001); I² = 92%							
Test for overall effect: Z = 3.49 (P = 0.0005)							
**Total (95% CI)**		**5047**		**5929**	**100.0%**	**1.83 [1.39, 2.41]**	
Total events	2603		1954				
Heterogeneity: Tau² = 0.30; Chi² = 154.34, df = 17 (P < 0.00001); I² = 89%							
Test for overall effect: Z = 4.32 (P < 0.0001)							
Test for subgroup differences: Chi² = 1.88, df = 1 (P = 0.17), I² = 46.9%							

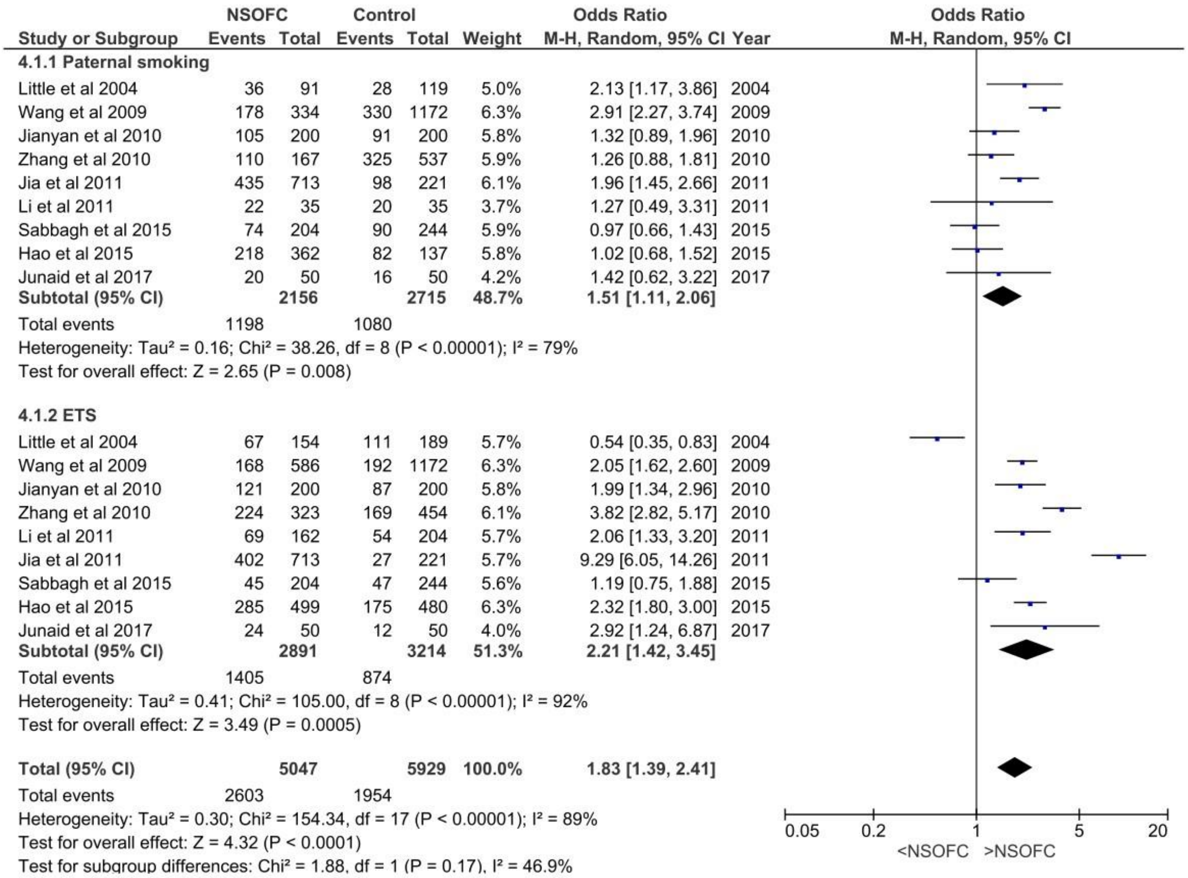

Supplementary Figure 5. Forest plot for meta-analysis of the association between the risk of having an infant NSOFC and its association with environmental tobacco smoking sub-grouped according to risk of bias.

Study or Subgroup	NSOFC Events	NSOFC Total	Control Events	Control Total	Weight	Odds Ratio M-H, Random, 95% CI	Year
**6.1.1 Low risk**							
Beaty et al 2001	24	107	18	130	2.9%	1.80 [0.92, 3.53]	2001
Little et al 2004	67	154	111	189	3.8%	0.54 [0.35, 0.83]	2004
Honein et al 2007	235	1227	554	2699	4.6%	0.92 [0.77, 1.09]	2007
Lie et al 2008	90	334	106	520	4.2%	1.44 [1.04, 1.99]	2008
Chevrier et al 2008	97	173	70	167	3.8%	1.77 [1.15, 2.72]	2008
Leite and Koifman 2009	166	274	281	548	4.3%	1.46 [1.09, 1.96]	2009
Wang et al 2009	168	586	192	1172	4.5%	2.05 [1.62, 2.60]	2009
Li et al 2010	59	88	348	651	3.6%	1.77 [1.11, 2.84]	2010
Jianyan et al 2010	121	200	87	200	3.9%	1.99 [1.34, 2.96]	2010
Mirilas et al 2011	34	35	25	35	0.6%	13.60 [1.63, 113.25]	2011
Taghavi et al 2012	113	300	80	300	4.1%	1.66 [1.18, 2.35]	2012
Sabbagh et al 2015	45	204	47	244	3.7%	1.19 [0.75, 1.88]	2015
Kummet et al 2016	1312	4508	2310	9626	4.8%	1.30 [1.20, 1.41]	2016
**Subtotal (95% CI)**		**8190**		**16481**	**48.7%**	**1.42 [1.17, 1.71]**	
Total events	2531		4229				
Heterogeneity: Tau² = 0.08; Chi² = 62.96, df = 12 (P < 0.00001); I² = 81%							
Test for overall effect: Z = 3.63 (P = 0.0003)							
**6.1.2 High risk**							
Zhang et al 2010	224	323	169	454	4.2%	3.82 [2.82, 5.17]	2010
Jia et al 2011	402	713	27	221	3.8%	9.29 [6.05, 14.26]	2011
Li et al 2011	69	162	54	204	3.7%	2.06 [1.33, 3.20]	2011
Hao et al 2015	285	499	175	480	4.4%	2.32 [1.80, 3.00]	2015
Hoyt et al 2016	148	1102	369	3324	4.6%	1.24 [1.01, 1.52]	2016
Mckinney et al 2016	41	95	20	95	3.0%	2.85 [1.50, 5.39]	2016
Dien et al 2017	67	170	43	170	3.7%	1.92 [1.21, 3.05]	2017
Goveas et al 2017	74	125	53	125	3.5%	1.97 [1.19, 3.26]	2017
Junaid et al 2017	24	50	12	50	2.3%	2.92 [1.24, 6.87]	2017
Pi et al 2018	140	240	664	1420	4.3%	1.59 [1.21, 2.10]	2018
Pi et al 2018 b	56	101	173	561	3.8%	2.79 [1.81, 4.30]	2018
Altoe et al 2019	32	150	38	300	3.4%	1.87 [1.11, 3.14]	2019
Chowchuen et al 2020	14	35	24	70	2.3%	1.28 [0.55, 2.95]	2020
Sato et al 2021	98	187	46566	94174	4.3%	1.13 [0.84, 1.50]	2021
**Subtotal (95% CI)**		**3952**		**101648**	**51.3%**	**2.23 [1.65, 3.01]**	
Total events	1674		48387				
Heterogeneity: Tau² = 0.27; Chi² = 112.66, df = 13 (P < 0.00001); I² = 88%							
Test for overall effect: Z = 5.24 (P < 0.00001)							
**Total (95% CI)**		**12142**		**118129**	**100.0%**	**1.80 [1.51, 2.15]**	
Total events	4205		52616				
Heterogeneity: Tau² = 0.17; Chi² = 224.93, df = 26 (P < 0.00001); I² = 88%							
Test for overall effect: Z = 6.46 (P < 0.00001)							
Test for subgroup differences: Chi² = 6.27, df = 1 (P = 0.01), I² = 84.0%							

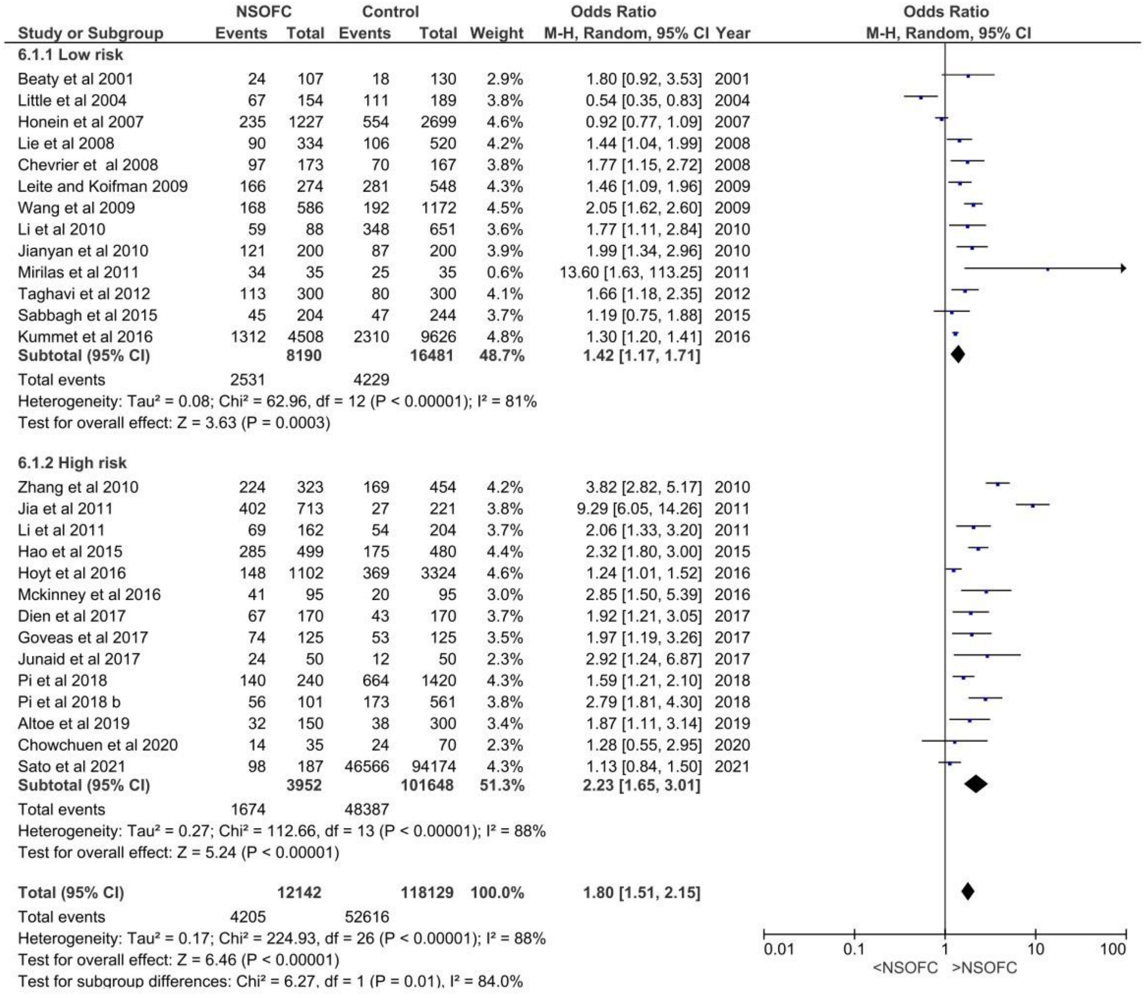

Supplementary Figure 6. Forest plot for meta-analysis of the association between maternal environmental tobacco smoking and the risk of having an infant with cleft lip and palate or cleft lip and its association with environmental tobacco smoking after excluding Jia et al, 2011 and Mirilas et al. 2011

Study or Subgroup	Experimental Events	Experimental Total	Control Events	Control Total	Weight	Odds Ratio M-H, Random, 95% CI
**5.1.1 CL/P**						
Beaty et al 2001	14	73	18	130	2.2%	1.48 [0.69, 3.18]
Chevrier et al 2008	65	119	70	167	3.5%	1.67 [1.04, 2.68]
Chowchuen et al 2020	14	35	24	70	1.9%	1.28 [0.55, 2.95]
Goveas et al 2017	74	125	53	125	3.3%	1.97 [1.19, 3.26]
Hao et al 2015	214	362	175	480	4.6%	2.52 [1.90, 3.33]
Honein et al 2007	147	699	554	2699	4.9%	1.03 [0.84, 1.27]
Hoyt et al 2016	101	740	369	3324	4.8%	1.27 [1.00, 1.60]
Jia et al 2011	302	537	27	221		Not estimable
Jianyan et al 2010	121	200	87	200	3.9%	1.99 [1.34, 2.96]
Leite and Koifman 2009	124	208	281	548	4.3%	1.40 [1.01, 1.94]
Lie et al 2008	58	210	106	520	4.0%	1.49 [1.03, 2.16]
Li et al 2010	59	88	348	651	3.5%	1.77 [1.11, 2.84]
Little et al 2004	40	76	111	189	3.1%	0.78 [0.46, 1.33]
Mckinney et al 2016	41	95	20	95	2.7%	2.85 [1.50, 5.39]
Mirilas et al 2011	34	35	25	35		Not estimable
Pi et al 2018	131	225	664	1420	4.5%	1.59 [1.19, 2.11]
Pi et al 2018 b	56	101	173	561	3.7%	2.79 [1.81, 4.30]
Sabbagh et al 2015	31	151	47	244	3.3%	1.08 [0.65, 1.80]
Sato et al 2021	82	146	46566	94174	4.3%	1.31 [0.94, 1.82]
Taghavi et al 2012	113	300	80	300	4.2%	1.66 [1.18, 2.35]
Zhang et al 2010	175	246	169	454		Not estimable
**Subtotal (95% CI)**		**3953**		**105897**	**66.6%**	**1.57 [1.33, 1.84]**
Total events	1485		49746			
Heterogeneity: Tau² = 0.07; Chi² = 52.38, df = 17 (P < 0.0001); I² = 68%						
Test for overall effect: Z = 5.51 (P < 0.00001)						
**5.1.2 CP**						
Beaty et al 2001	10	34	18	133	1.8%	2.66 [1.09, 6.48]
Chevrier et al 2008	32	54	70	167	2.7%	2.02 [1.08, 3.76]
Hao et al 2015	71	137	175	480	4.0%	1.87 [1.28, 2.75]
Honein et al 2007	88	528	554	2699	4.7%	0.77 [0.60, 0.99]
Hoyt et al 2016	47	362	369	3324	4.3%	1.19 [0.86, 1.65]
Jia et al 2011	100	176	27	221		Not estimable
Lie et al 2008	32	124	106	520	3.6%	1.36 [0.86, 2.14]
Little et al 2004	27	42	111	189	2.4%	1.26 [0.63, 2.53]
Pi et al 2018	9	15	664	1420	1.4%	1.71 [0.60, 4.82]
Sabbagh et al 2015	14	53	47	244	2.5%	1.50 [0.76, 3.00]
Sato et al 2021	16	41	46566	94174	2.7%	0.65 [0.35, 1.23]
Zhang et al 2010	49	77	169	454	3.3%	2.95 [1.79, 4.87]
**Subtotal (95% CI)**		**1467**		**103804**	**33.4%**	**1.44 [1.06, 1.94]**
Total events	395		48849			
Heterogeneity: Tau² = 0.18; Chi² = 39.93, df = 10 (P < 0.0001); I² = 75%						
Test for overall effect: Z = 2.34 (P = 0.02)						
**Total (95% CI)**		**5420**		**209701**	**100.0%**	**1.51 [1.31, 1.75]**
Total events	1880		98595			
Heterogeneity: Tau² = 0.10; Chi² = 97.40, df = 28 (P < 0.00001); I² = 71%						
Test for overall effect: Z = 5.61 (P < 0.00001)						
Test for subgroup differences: Chi² = 0.24, df = 1 (P = 0.62), I² = 0%						

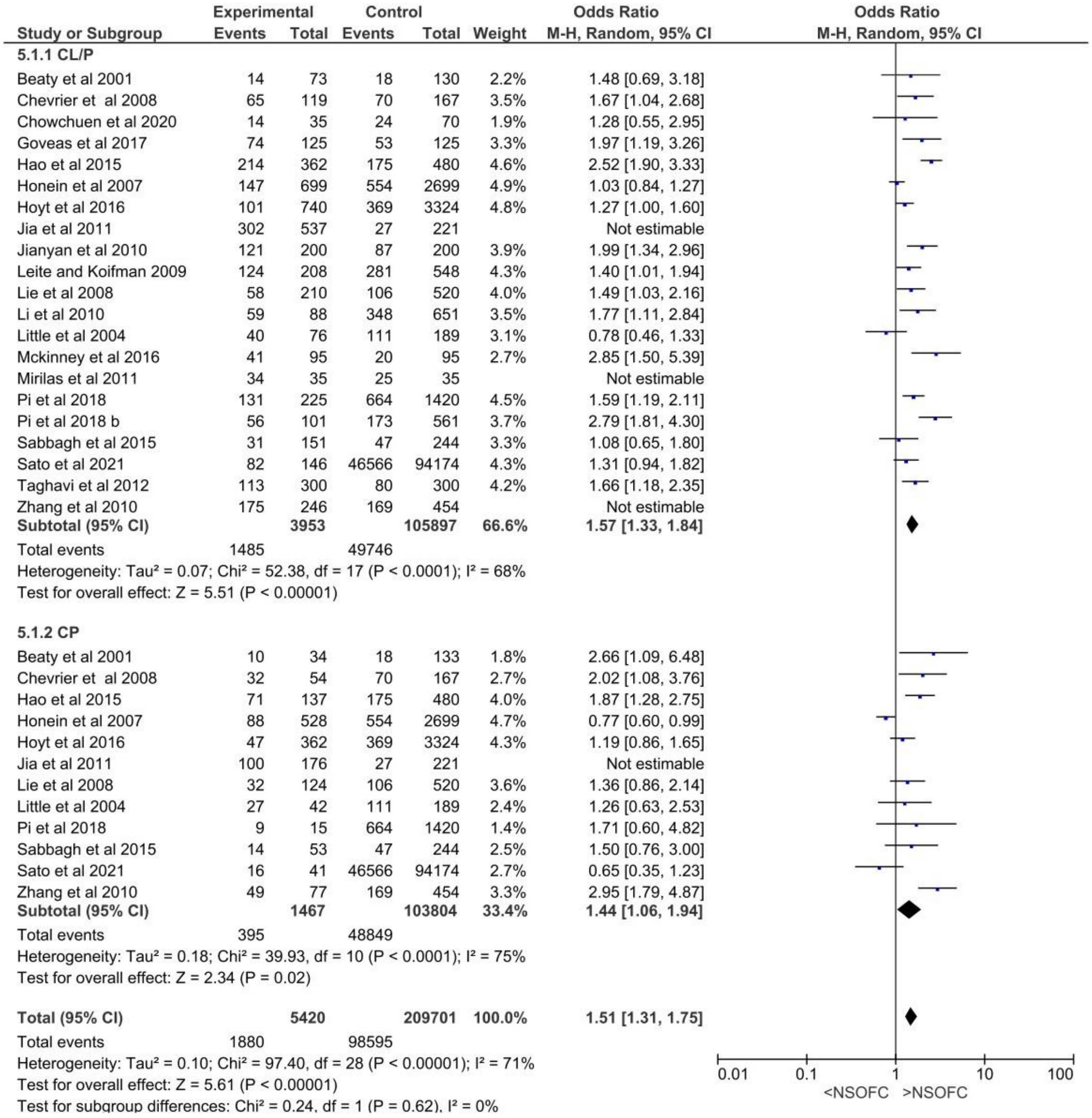

Supplementary Figure 7. Forest plot for meta-analysis of the association between maternal environmental tobacco smoking and the risk of having an infant with cleft lip and palate or cleft lip and its association with environmental tobacco smoking using the reported adjusted odds ratio.

Study or Subgroup	log[Odds Ratio]	SE	Weight	Odds Ratio IV, Random, 95% CI	Year
**7.2.1 CL/P**					
Beaty et al 2001	0.0392	0.2243	3.9%	1.04 [0.67, 1.61]	2001
Little et al 2004	-0.1054	0.2731	3.4%	0.90 [0.53, 1.54]	2004
Honein et al 2007	0.0306	0.1043	4.8%	1.03 [0.84, 1.26]	2007
Lie et al 2008	0.399	0.1889	4.2%	1.49 [1.03, 2.16]	2008
Chevrier et al 2008	0.5878	0.2731	3.4%	1.80 [1.05, 3.07]	2008
Wang et al 2009	0.7178	0.143	4.6%	2.05 [1.55, 2.71]	2009
Leite and Koifman 2009	0.3293	0.1629	4.4%	1.39 [1.01, 1.91]	2009
Li et al 2010	0.6931	0.2606	3.5%	2.00 [1.20, 3.33]	2010
Jianyan et al 2010	0.6878	0.2031	4.1%	1.99 [1.34, 2.96]	2010
Jia et al 2011	2.2225	0.2232	3.9%	9.23 [5.96, 14.30]	2011
Mirilas et al 2011	0.5933	0.492	2.0%	1.81 [0.69, 4.75]	2011
Taghavi et al 2012	-0.4894	0.1809	4.2%	0.61 [0.43, 0.87]	2012
Sabbagh et al 2015	-0.1474	0.2612	3.5%	0.86 [0.52, 1.44]	2015
Hao et al 2015	0.9243	0.1429	4.6%	2.52 [1.90, 3.33]	2015
Kummet et al 2016	0.1044	0.0584	5.1%	1.11 [0.99, 1.24]	2016
Hoyt et al 2016	0.2357	0.1205	4.7%	1.27 [1.00, 1.60]	2016
**Subtotal (95% CI)**			**64.3%**	**1.52 [1.18, 1.95]**	
Heterogeneity: Tau² = 0.22; Chi² = 152.95, df = 15 (P < 0.00001); I² = 90%					
Test for overall effect: Z = 3.25 (P = 0.001)					
**7.2.2 CP**					
Beaty et al 2001	0.157	0.2769	3.4%	1.17 [0.68, 2.01]	2001
Little et al 2004	0.0953	0.4023	2.5%	1.10 [0.50, 2.42]	2004
Honein et al 2007	0.0953	0.1625	4.4%	1.10 [0.80, 1.51]	2007
Chevrier et al 2008	0.7706	0.3172	3.1%	2.16 [1.16, 4.02]	2008
Lie et al 2008	0.0488	0.3299	3.0%	1.05 [0.55, 2.00]	2008
Leite and Koifman 2009	0.5128	0.3154	3.1%	1.67 [0.90, 3.10]	2009
Hao et al 2015	0.6286	0.1955	4.1%	1.87 [1.28, 2.75]	2015
Sabbagh et al 2015	0.4086	0.3513	2.8%	1.50 [0.76, 3.00]	2015
Kummet et al 2016	0.1655	0.0844	5.0%	1.18 [1.00, 1.39]	2016
Hoyt et al 2016	0.178	0.1658	4.4%	1.19 [0.86, 1.65]	2016
**Subtotal (95% CI)**			**35.7%**	**1.28 [1.12, 1.46]**	
Heterogeneity: Tau² = 0.00; Chi² = 9.98, df = 9 (P = 0.35); I² = 10%					
Test for overall effect: Z = 3.72 (P = 0.0002)					
**Total (95% CI)**			**100.0%**	**1.45 [1.22, 1.72]**	
Heterogeneity: Tau² = 0.15; Chi² = 163.46, df = 25 (P < 0.00001); I² = 85%					
Test for overall effect: Z = 4.27 (P < 0.0001)					
Test for subgroup differences: Chi² = 1.40, df = 1 (P = 0.24), I² = 28.6%					

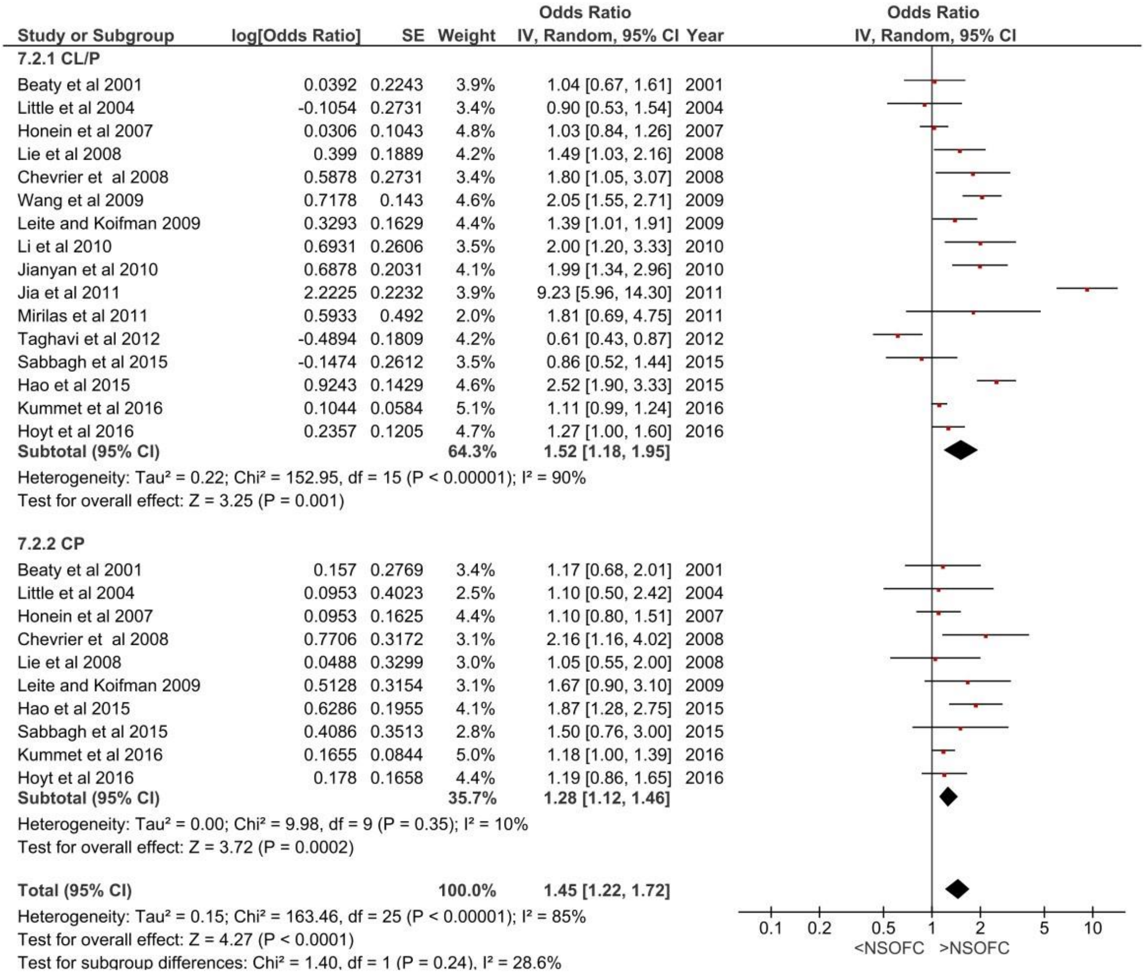

Supplementary Figure 8. Forest plot for meta-analysis of the association between maternal environmental tobacco smoking and the risk of having an infant with NSOFC according to period of exposure

Study or Subgroup	NSOFC Events	NSOFC Total	Control Events	Control Total	Weight	Odds Ratio M-H, Random, 95% CI
**5.4.1 First trimester period**						
Little et al 2004	67	154	111	189	5.5%	0.54 [0.35, 0.83]
Sato et al 2021	98	187	46566	94174	6.2%	1.13 [0.84, 1.50]
Kummet et al 2016	1312	4508	2310	9626	6.9%	1.30 [1.20, 1.41]
Lie et al 2008	90	334	106	520	6.1%	1.44 [1.04, 1.99]
Chevrier et al 2008	97	173	70	167	5.5%	1.77 [1.15, 2.72]
Beaty et al 2001	24	107	18	130	4.2%	1.80 [0.92, 3.53]
Altoe et al 2019	32	150	38	300	5.0%	1.87 [1.11, 3.14]
Jia et al 2011	402	713	27	221	5.5%	9.29 [6.05, 14.26]
**Subtotal (95% CI)**		**6326**		**105327**	**44.8%**	**1.66 [1.09, 2.53]**
Total events	2122		49246			
Heterogeneity: Tau² = 0.32; Chi² = 101.77, df = 7 (P < 0.00001); I² = 93%						
Test for overall effect: Z = 2.36 (P = 0.02)						
**5.4.2 Pregestation and First trimester periods**						
Honein et al 2007	235	1227	554	2699	6.7%	0.92 [0.77, 1.09]
Sabbagh et al 2015	45	204	47	244	5.3%	1.19 [0.75, 1.88]
Hoyt et al 2016	148	1102	369	3324	6.6%	1.24 [1.01, 1.52]
Chowchuen et al 2020	14	35	24	70	3.4%	1.28 [0.55, 2.95]
Li et al 2010	59	88	348	651	5.2%	1.77 [1.11, 2.84]
Dien et al 2017	67	170	43	170	5.3%	1.92 [1.21, 3.05]
Jianyan et al 2010	121	200	87	200	5.7%	1.99 [1.34, 2.96]
Li et al 2011	69	162	54	204	5.4%	2.06 [1.33, 3.20]
Hao et al 2015	285	499	175	480	6.4%	2.32 [1.80, 3.00]
Mckinney et al 2016	41	95	20	95	4.3%	2.85 [1.50, 5.39]
Mirilas et al 2011	34	35	25	35	0.9%	13.60 [1.63, 113.25]
**Subtotal (95% CI)**		**3817**		**8172**	**55.2%**	**1.69 [1.28, 2.22]**
Total events	1118		1746			
Heterogeneity: Tau² = 0.15; Chi² = 58.25, df = 10 (P < 0.00001); I² = 83%						
Test for overall effect: Z = 3.71 (P = 0.0002)						
**Total (95% CI)**		**10143**		**113499**	**100.0%**	**1.67 [1.35, 2.07]**
Total events	3240		50992			
Heterogeneity: Tau² = 0.17; Chi² = 159.95, df = 18 (P < 0.00001); I² = 89%						
Test for overall effect: Z = 4.68 (P < 0.00001)						
Test for subgroup differences: Chi² = 0.00, df = 1 (P = 0.95), I² = 0%						

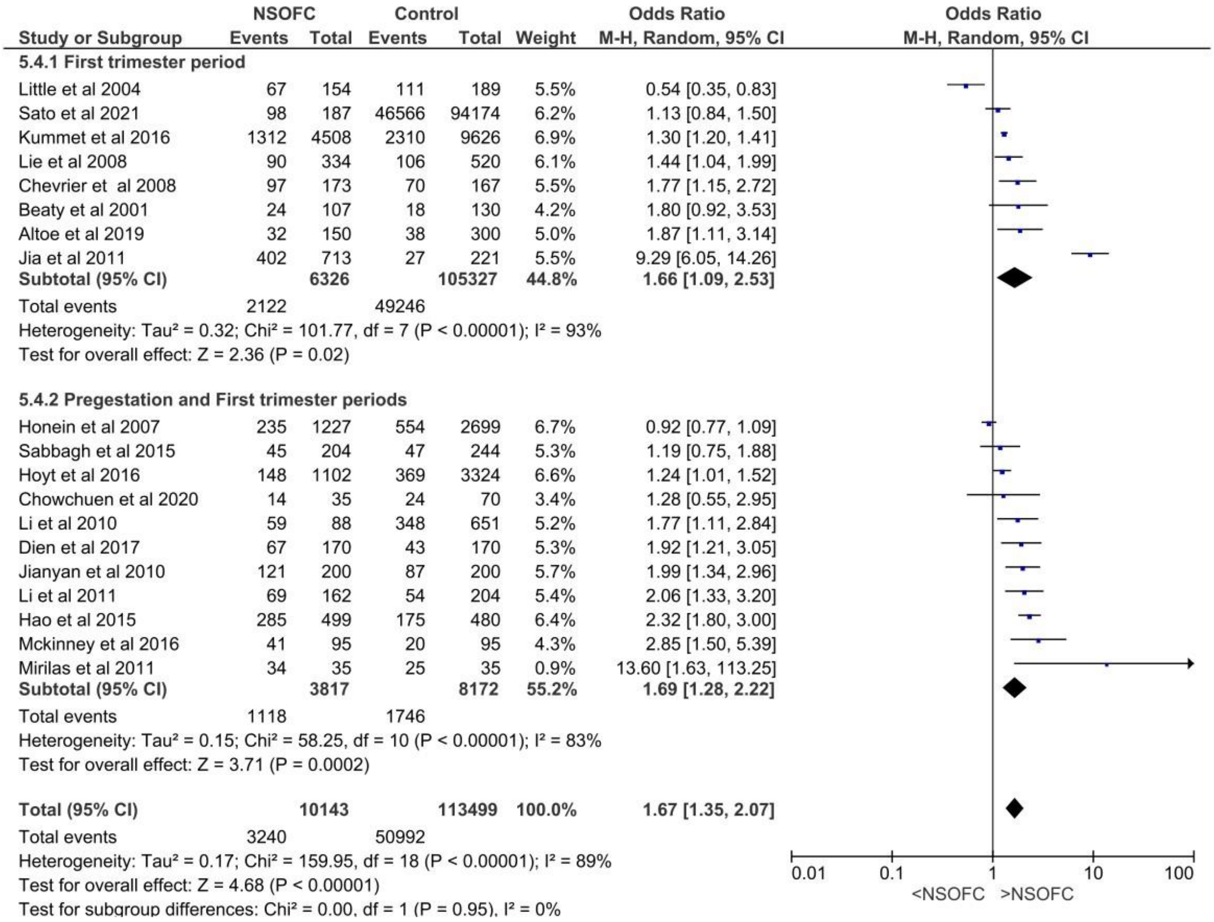

Supplementary Figure 9. Funnel plot for studies showing the relationship between maternal environmental tobacco exposure and both CL/P and CP.

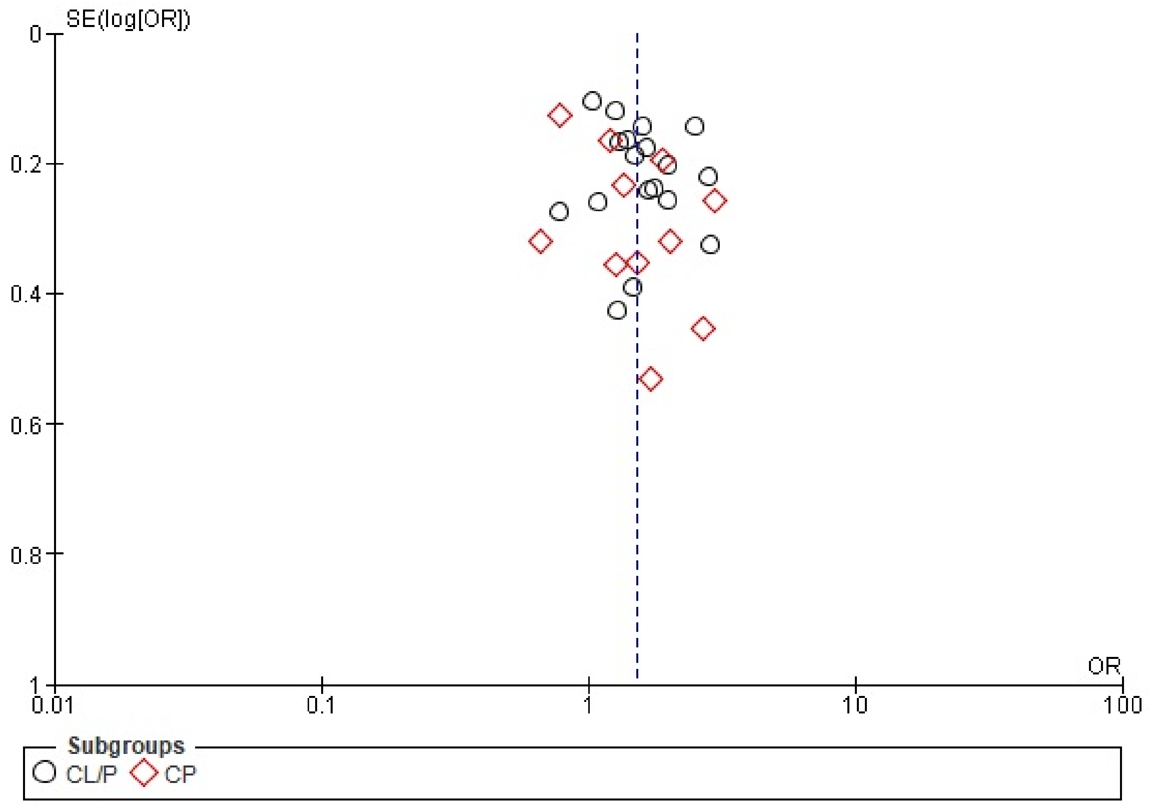

Supplementary Figure 10. Funnel plot for studies showing the relationship between maternal environmental tobacco exposure and NSOFC for both low risk and high risk studies.

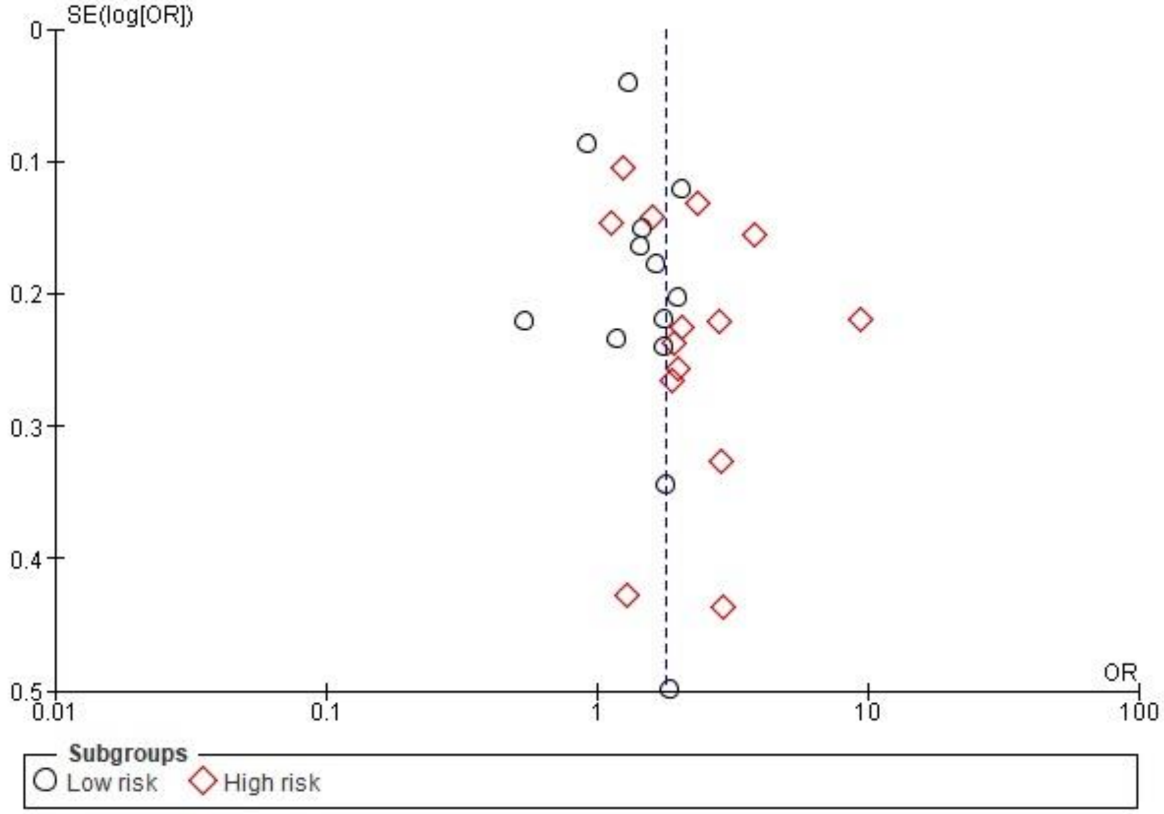

Supplementary Figure 11. Cumulative meta-analysis for the stability of evidence from 2011 through 2021.

Cumulative Meta Analysis

Study name	Cumulative statistics				
	Point	Lower limit	Upper limit	Z-Value	p-Value
Beaty et al 2001	1.799	0.917	3.530	1.708	0.088
Little et al 2004	0.958	0.296	3.106	-0.071	0.944
Honein et al 2007	0.908	0.549	1.501	-0.377	0.706
Chevrier et al 2008	1.080	0.670	1.739	0.315	0.753
Lie et al 2008	1.144	0.779	1.682	0.687	0.492
Wang et al 2009	1.281	0.857	1.915	1.209	0.227
Leite and Koifman 20[illegible]	[illegible]906	0.931	1.832	1.547	0.122
Zhang et al 2010	1.503	1.007	2.244	1.993	0.046
Jianyan et al 2010	1.550	1.074	2.237	2.342	0.019
Li et al 2010	1.570	1.118	2.205	2.601	0.009
Mirilas et al 2011	1.583	1.142	2.196	2.754	0.006
Jia et al 2011	1.840	1.247	2.716	3.071	0.002
Li et al 2011	1.856	1.289	2.673	3.323	0.001
Taghavi et al 2012	1.840	1.314	2.578	3.545	0.000
Sabbagh et al 2015	1.788	1.298	2.462	3.558	0.000
Hao et al 2015	1.819	1.351	2.451	3.937	0.000
Mckinney et al 2016	1.861	1.394	2.485	4.214	0.000
Kummet et al 2016	1.814	1.432	2.298	4.937	0.000
Hoyt et al 2016	1.771	1.423	2.203	5.130	0.000
Dien et al 2017	1.777	1.439	2.195	5.336	0.000
Goveas et al 2017	1.785	1.454	2.191	5.536	0.000
Junaid et al 2017	1.810	1.479	2.216	5.748	0.000
Pi et al 2018	1.797	1.483	2.178	5.971	0.000
Pi et al 2018 b	1.827	1.512	2.208	6.246	0.000
Altoe et al 2019	1.828	1.521	2.199	6.413	0.000
Chowchuen et al 202[illegible]	[illegible].812	1.512	2.173	6.425	0.000
Sato et al 2021	1.775	1.490	2.115	6.427	0.000
	1.775	1.490	2.115	6.427	0.000

Cumulative odds ratio (95% CI)

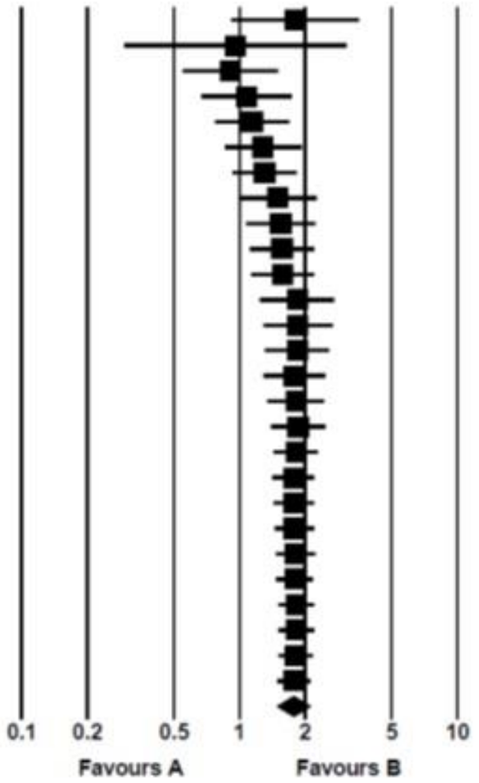

© 2023 Sabbagh H.J. et al.
